# Type 2 and Type 17 Invariant Natural Killer T Cells Contribute to Local Eosinophilic and Neutrophilic Inflammation and Their Function Is Regulated by Mucosal Microenvironment in Nasal Polyps

**DOI:** 10.3389/fimmu.2022.803097

**Published:** 2022-06-03

**Authors:** Xiaoyan Ye, Qing Bao, Hexin Chen, Qingxiang Meng, Qianying Li, Lin Sun, Jian Li, Wenbin Lei, Weiping Wen, Wenjing He, Linyi Jiao, Bixing Fang, Yifang Gao, Chunwei Li

**Affiliations:** ^1^ Department of Otolaryngology, Guangzhou Key Laboratory of Otorhinolaryngology, The First Affiliated Hospital, Sun Yat-sen University, Guangzhou, China; ^2^ Department of Ophthalmology, Zhongnan Hospital of Wuhan University, Wuhan, China; ^3^ Department of Otorhinolaryngology Head and Neck Surgery, Guangzhou First People’s Hospital, Guangzhou, China; ^4^ Organ Transplantation Centre, Guangdong Provincial Key Laboratory of Organ Donation and Transplant Immunology, The First Affiliated Hospital, Sun Yat-sen University, Guangzhou, China

**Keywords:** invariant natural killer T cells, functional subsets, chronic rhinosinusitis with nasal polyps (CRSwNP), eosinophilia, neutrophilia

## Abstract

Chronic rhinosinusitis with nasal polyps (CRSwNP) is characterized by heterogeneous inflammatory endotypes of unknown etiology. Invariant natural killer T (iNKT) cells are multifunctional innate T cells that exhibit Th1-, Th2-, and Th17-like characteristics. We investigated functional relationships between iNKT cells and inflammatory subtypes of CRSwNP. Eighty patients with CRSwNP and thirty-two control subjects were recruited in this study. Flow cytometry was used to analyze the frequencies and functions of iNKT cells and their subsets in peripheral blood mononuclear cells (PBMCs) and tissues. Polyp tissue homogenates were used to study the multifunctionality of iNKT cells. iNKT cells were significantly increased in polyps (0.41%) than in control mucosa (0.12%). iNKT cells were determined in the paucigranunlocytic (n=20), eosinophilic (n=22), neutrophilic (n=23), and mixed granulocytic (n=13) phenotypes of CRSwNP. The percentages of iNKT cells and HLA-DR^+^PD-1^+^ subsets were lower in eosinophilic or mixed granulocytic polyps than those of other phenotypes. iNKT cells and subsets were enriched in polyp tissues than in matched PBMCs. The evaluation of surface markers, transcription factors, and signature cytokines indicated that the frequencies of iNKT2 and iNKT17 subsets were significantly increased in eosinophilic and neutrophilic polyps, respectively, than in the paucigranulocytic group. Moreover, the production of type 2 (partially dependent on IL-7) and type 17 (partially dependent on IL-23) iNKT cells could be stimulated by eosinophilic and neutrophilic homogenates, respectively. Our study revealed that type 2 and type 17 iNKT cells were involved in eosinophilic and neutrophilic inflammation, respectively, in CRSwNP, while different inflammatory microenvironments could modulate the functions of iNKT cells, suggesting a role of iNKT cells in feedback mechanisms and local inflammation.

## Introduction

Chronic rhinosinusitis with nasal polyps (CRSwNP) is characterized by the infiltration of multiple inflammatory cells and tissue remodeling (e.g., edema) in the sinus mucosa. However, the etiology and pathogenesis of CRSwNP remain poorly understood due to the presence of heterogeneous pathological endotypes in the local environment (1, 2). Polyps showing eosinophilia with an increased Th2 response (e.g., increase of IL-4 and IL-5 levels) is the dominant inflammatory endotype in Caucasian patients (3), while a high proportion of neutrophilic polyps is found in Asian patients (4). Based on the levels of dominant eosinophils and/or neutrophils, chronic airway inflammation (such as asthma and CRSwNP) can be classified into four inflammatory subtypes, i.e., paucigranulocytic, eosinophilic, neutrophilic, and mixed granulocytic (5, 6). It is well known that Th2 cytokines contribute to eosinophilic inflammation, while IL-17A is more involved to a greater extent in the neutrophilic inflammation of both the upper and lower airway mucosae (7). Consequently, the mechanisms underlying the effects of these inflammatory subtypes in chronic airway inflammation, including CRSwNP, might be distinct.

Invariant natural killer T (iNKT) cells are a type of innate T cells that express an invariant T cell receptor (TCR) (Vα24-Jα18 in humans, Vα14-Jα18 in mice) and recognize lipid antigens *via* an MHC-like molecule CD1d (8). α-Galactosylceramide (αGalCer) is one of the most potent antigens that activates iNKT cells (8). iNKT cells generally develop in the thymus, and matured iNKT cells later move into the peripheral tissues (9, 10). iNKT cells are innate T lymphocytes. They respond rapidly to TCR signals from pathogens or tissue microenvironments to produce effector cytokines, including IFN-γ, IL-4, IL-5, IL-13, and IL-17A (11). Such a rapid response causes iNKT cells to serve as a bridge that links innate and adaptive immune responses in local tissues (12). There are several functional subsets of iNKT cells having Th1-, Th2-, or Th17-like properties, such as type 1 (iNKT1), type 2 (iNKT2), and type 17 (iNKT17), characterized by transcription factors, surface markers, and cytokine production (8, 13). iNKT cells also play important roles in the development of chronic inflammation (8, 14), e.g., accumulation of iNKT cells in hepatic tissues would contribute fibrosis progression in non-alcoholic fatty liver disease (15), while in a hepatitis B virus transgenic mouse model, iNKT cells could induce the activation of hepatic stellate cells though IL-4 and IL-13 production and accelerate liver fibrosis (16).

Most of the studies related to iNKT cells in chronic airway inflammation have been carried out using murine models. Although iNKT cells can protect against lung infections by bacteria (17), the activation of iNKT cells by αGalCer caused massive eosinophilia *via* the production of IL-4, IL-5, and IL-13 in an OVA-induced allergic rhinitis mouse model; such cells could also promote the infiltration of neutrophils *via* IL-17A production (18–21). A study reported that iNKT cells were frequently found in asthmatic patients, and up to 2% of the total T cells were iNKT cells in bronchoalveolar lavage fluid (BALF) (22). However, due to the multifunctional property of iNKT cells, the frequency alone does not represent the severity of asthma. Furthermore, αGalCer is a highly potent antigen that activates iNKT functions, and iNKT functions found in murine models may not reflect the pathological patterns in humans. Thus, the relationship between iNKT cells and the pathophysiology of chronic airway inflammation in humans remains elusive.

Generally, chronic inflammation in the upper airway and lower airway mucosa shares similar histopathologic features, including eosinophil infiltration and an enhanced Th2 response. A high proportion of patients with CRSwNP also suffer from asthmatic symptoms. To date, two relevant studies have reported iNKT cells in CRS. Yamamoto et al. showed that the mRNA transcript of iNKT cell–specific surface marker (TCRVα24) was detected in eosinophilic CRS tissues but not in neutrophilic CRS tissues (23); Fereidouni et al. used flow cytometry and qPCR assays to determine a higher percentage of iNKT cells in polyp tissues than in matched PBMCs from the same patients (24). However, these studies did not indicate any subsets of iNKT cells or their relationship with different inflammatory endotypes. An important characteristic of iNKT cells is their multifunctionality; hence, characterizing different functional subsets of iNKT cells is important to reveal the roles of iNKT cells in mucosal inflammation. We sought to investigate the roles of iNKT cells in CRSwNP pathogenesis by considering the following two aspects: 1) association of frequencies and functional subsets of iNKT cells with inflammatory subtypes of CRSwNP and 2) effect of the inflammatory microenvironment of CRSwNP in regulating the functional development of iNKT cells.

## Materials and Methods

### Study Subjects and Sample Collection

CRSwNP patients (n=80) were recruited from the First Affiliated Hospital, Sun Yat-sen University, Guangzhou, China. Patients with CRSwNP were diagnosed by an ENT physician by assessing symptoms (nasal blockage, obstruction, congestion, or nasal discharge), endoscopy signs (visible polyps in middle meatus, edematous sinus mucosa, mucopurulent discharge primarily from middle meatus), and CT scan (mucosal changes within sinuses). Polyp tissues were obtained from patients with CRSwNP who underwent functional endoscopic sinus surgery. In addition, peripheral blood samples were collected from 33 CRSwNP patients and healthy volunteers (aged 24–59 years; n=15). The tissues of biopsy samples obtained from the middle turbinate mucosa from subjects (n=32) without rhinosinusitis (diagnosed as nasal fracture or nasal septal deviation) who were scheduled for septal surgery served as controls. None of the control subjects had allergic symptoms or other chronic nasal inflammation of the sinus or nasal cavities. The atopy status for each case was evaluated by an allergen test, using an ImmunoCap assay (Phadia, Uppsala, Sweden) to measure serum-specific IgE (sIgE) to eight common inhalant allergens, including *Dermatophagoides pteronyssinus, D. farina, cat dander, dog dander, German cockroach, Alternaria alternata, common ragweed, and mugwort*. The sIgE value of more than 0.35 kUA/L was considered a positive allergic response. None of the CRSwNP patients and control subjects had immunodeficiencies, autoimmune diseases, tumor, chronic obstructive pulmonary disease, vasculitis, other organ-related diseases, or acute respiratory tract infection 1 month before inclusion. None of the subjects were administered systemic glucocorticoid steroids, antibiotics 3 months, and/or nasal corticosteroids 1 month before the surgery. The clinical and pathological characteristics of patients and controls are shown in **Table 1**. The materials used for different experimental assays are described in **Supplementary Table 1**.

**Table 1:** Clinical and histopathological characteristics of patients and control subjects.

Clinical and histopathological factors	Controls	CRSwNP	P-value
Gender (male/female)	20/12	55/25	NS
Age (median)	46	41	NS
Atopy	0	11	NA
Asthma	0	6	NA
Smoker	3	11	NA
Recurrence	0	13	NA
Eosinophils (median, 1st and 3rd quartile)	1 (1, 4)	9 (3, 26)	<0.001
Neutrophils (median, 1st and 3rd quartile)	2 (1, 3)	10 (2, 23)	<0.001
Pauciguanulocytic	30	20	NA
Eosinophilic	1	23	NA
Neutrophilic	1	24	NA
Mixed granulocytic	0	13	NA

NS, not significant; NA, not applicable.

### Sample Processing Procedures

Fresh tissue specimens were processed using the following methods: i) formalin-fixed paraffin embedding (FFPE), ii) the isolation of local tissue cells, and iii) tissue homogenization. PBMCs were isolated from peripheral blood samples.

#### Cell Isolation From Peripheral Blood and Tissues

PBMCs from healthy subjects and patients with CRSwNP were isolated by Ficoll-Hypaque (MP Biomedicals, Santa Ana, CA, United States) density gradient centrifugation within 24 h of blood collection. The tissues obtained from the control nasal mucosa and polyp tissues were cut into small pieces, and digested in an RPMI 1640 medium with collagenase I for 1 h at 37°C. A single-cell suspension was obtained by filtering the digested tissue fragments through a 100 μm cell nylon mesh (CORNING, Corning, NY, United States). Both PBMCs and tissue-isolated cells were aliquoted and stored with a freezing medium (90% fetus bovine serum plus 10% dimethyl sulfoxide) in liquid nitrogen until use. A portion of freshly isolated tissue cells from some tissue samples were subjected to Ficoll-Hypaque density gradient centrifugation to obtain mononuclear cells for intracellular cytokine examination.

#### Preparation of Tissue Homogenates

TheFresh tissues from patients with polyps were cut into pieces and homogenized on ice for 3 min in phosphate buffered saline (PBS) buffer containing a protease/phosphor-protease inhibitor cocktail (Keygentec, Nanjing, China) using Bullet Blender Blue equipment (Next Advance, Averill Park, NY, United States). The supernatant thus obtained was centrifuged at 4°C and stored at -80°C until use.

### Histochemical and Immunohistochemical Staining Experiment

The FFPE samples were cut into 4 μm thick sections to perform histological and immunohistochemical staining. Eosinophils were examined by hematoxylin and eosin (H&E) staining. The FFPE sections were pretreated with target retrieval buffer (PH6) (Dako, Glostrup, Denmark) and stained with a mouse anti-human neutrophil elastase monoclonal antibody (Abcam, Cambridge, MA, United States) at 4°C overnight. Next, the tissue sections were incubated with DAKO EnVision+System-HRP (Dako, Glostrup, Denmark) at room temperature for 30 min, stained with diaminobenzidine substrate (Dako, Glostrup, Denmark) for color development, and counterstained with hematoxylin.

### Evaluation of Inflammatory Phenotypes in Polyp Tissues

The number of eosinophils or neutrophils was counted in three randomly selected microscopic fields in each tissue section at ×400 magnification. The tissue sections were coded by the number, and the researcher independently observed all cases. Based on the local infiltration level of eosinophils and neutrophils in each high-power field, four inflammatory phenotypes were defined based on a modification of previous criteria: eosinophilic phenotype was defined as >10 eosinophils (25), a neutrophilic phenotype was defined as >10 neutrophils (26), a paucigranulocytic phenotype was defined as ≤10 eosinophils and ≤10 neutrophils, and a mixed granulocytic phenotype was defined as >10 eosinophils and >10 neutrophils.

### Flow Cytometry Analysis of iNKT Cells and Subsets

Flow cytometry analysis was performed in PBMCs from healthy donors and patients, and isolated tissue cells from control subjects and CRSwNP patients. The antibodies used for flow cytometry are listed in **Supplementary Table 2**. Antibodies were purchased from Biolegend (San Diego, CA, United States), BD Bioscience (San Jose, CA, United States), Milteny Biotec (Bergisch Gladbach, Germany), and Thermo Fisher Scientific (Waltham, MA, United States).

To characterize total iNKT cells and iNKT subsets, the PBMCs and isolated tissue cells were directly stained with primary antibodies targeting different surface markers at room temperature for 15 min in the dark. The panel of markers used for detecting total iNKT cells and the activation/exhaustion status of iNKT cells consisted of CD3, CD4, TCR Vα24, PD-1, and HLA-DR; another panel of markers for detecting functional iNKT subsets comprised CD3, CD4, TCR Vα24, CXCR3, CCR4, and CCR6. Due to a small number of iNKT cells in tissues and PBMCs, in order to gain an accurate analysis, the event number was maximized when running a flow cytometry experiment.

To analyze intracellular cytokine production in iNKT cells, mononuclear cells from tissues were first expanded by using IL-2 (100 IU/ml) plus αGalCer (AbMole BioScience, Houston, TX, United States) (100 ng/ml) in complete an RPMI1640 medium for 10 days based on published methods (27). The cells were incubated with Cell Stimulation Cocktail plus Protein Transport Inhibitors (containing Phorbol 12-myristate 13-acetate (PMA) and Brefeldin A (BFA)) (Thermo Fisher Scientific) for 6 h in a CO_2_ incubator, and the cells were stained with conjugated primary antibodies for CD4 and TCR Vα24 followed by fixation/permeabilization. Finally, the cells were incubated with conjugated primary antibodies for CD3, IFN-γ, IL-5, IL-13, and IL-17A. The stained cells were analyzed using a BD Fortessa X20 flow cytometer (BD Bioscience). Flow cytometry data were analyzed by using FlowJo software (Treestar, San Carlos, CA, United States).

### iNKT Cell Sorting, RNA Extraction, and Quantitative Rt-Pcr Experiment

Frozen tissue cells were recovered and stained with conjugated antibodies targeting CD3 and TCR Vα24; iNKT cells were then isolated by BD FACS Aria II (BD Bioscience, San Jose, CA, United States), and the sorted iNKT cells were stimulated with PMA/Inomycin. Total RNA was extracted from the stimulated cells by using an EZ-press SINGLE Cell to cDNA kit (EZBioscience, Roseville, MN), which could facilitate RNA extraction from low cell numbers. Briefly, the cDNA was pre-amplified using gene-specific primers (Glyceraldehyde-3-Phosphate Dehydrogenase (GAPDH), T-bet, GATA Binding Protein 3 (GATA3), and RAR Related Orphan Receptor C (RORc)) from the cell lysate, and then, the pre-amplification product was analyzed by quantitative PCR employing the SYBR green assay (Roche, Basel, Switzerland). The relative expression levels of the target genes were normalized to those of GAPDH. The information related to the primers used and presented in **Supplementary Table 3**.

### PBMCs Treated With Tissue Homogenates and Neutralizing Antibodies

The total protein levels of all the tissue homogenates were measured, and the homogenates containing 500 ng/ml protein content were and added to PBMCs as stimulants. The PBMCs from healthy donors were incubated with IL-2 plus αGalCer in the presence of eosinophilic or neutrophilic polyp homogenates for 10 days in a CO_2_ incubator. PBMCs treated with eosinophilic and neutrophilic homogenates were also incubated with or without anti-IL-7- and anti-IL-23-neutralizing antibodies. The cells were harvested and restimulated with Cell Stimulation Cocktail plus Protein Transport Inhibitors (Thermo Fisher Scientific, Waltham, MA, United States). The cytokine production profiles of iNKT cells were analyzed using a BD Fortessa X20 flow cytometer (BD Bioscience, San Jose, CA, United States).

### ELISA

The supernatants of tissue homogenate or cell-free culture supernatants were harvested and evaluated by ELISA for IL-7, IL-23, and IL-15 production according to the manufacturers’ protocols. The ELISA kits were purchased from R&D Systems (Minnesota, MN, United States). The detection ranges for the above cytokines were listed below: IL-7 (7.8–500 pg/ml), IL-23 (125.0–8,000 pg/ml), and IL-15 (15.6–1,000 pg/ml).

### Statistical Analysis

The Mann–Whitney U test was used to compare differences in iNKT cells or subsets between two independent groups. The Spearman rank test was used to analyze the relationship between the percentages of iNKT cells or subsets and infiltration of eosinophils or neutrophils. The Wilcoxon signed-rank test was used to compare the differences between two dependent groups. Following statistical analyses, graphs were generated using GraphPadPrism version 8.0 (GraphPad Software, San Diego, CA, United States).

## Results

### Frequencies of iNKT Cells in PBMCs and Tissues From Patients With CRSwNP

**Figure 1:**
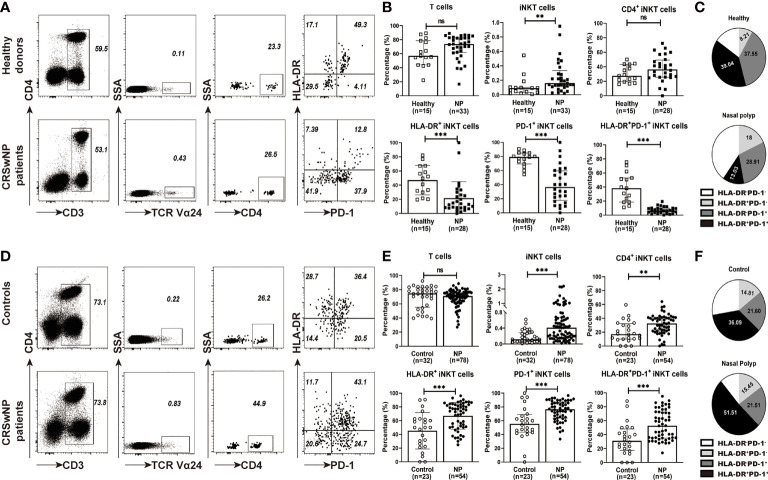
The percentages of iNKT cells and their subsets in PBMCs and tissues from CRSwNP and control (or healthy) subjects. **(A)** Representative flow cytometric plots showed the frequencies of iNKT cells, CD4^+^ iNKT cells, HLA-DR^+^ iNKT cells, PD-1^+^ iNKT cells, and HLA-DR^+^PD-1^+^ iNKT cells in PBMCs from healthy controls and patients. FACS analysis for stepwise iNKT cell enumeration is based on subgating T cells for CD3 and TCR Va24. **(B)** The percentages of T cells, iNKT cells and iNKT subsets in PBMCs were compared between healthy donors (n = 15) and CRSwNP patients (33 samples used for total T-cell and iNKT-cell analysis, 28 samples used for iNKT subset analysis). **(C)** The proportions of HLA-DR^+^PD-1^-^, HLA-DR^+^PD-1^+^, and HLA-DR^-^PD-1^-^ iNKT subsets in total iNKT cells from PBMCs (n = 15) of healthy and patient subjects (n = 28). **(D)** Representative flow cytometric plots showed the frequencies of iNKT cells, CD4+ iNKT cells, HLA-DR^+^ iNKT cells, PD-1^+^ iNKT cells, and HLA-DR^+^PD-1^+^ iNKT cells in nasal mucosa from control and patient subjects. The gating strategy is the same as stated in **(A)**. **(E)** The percentages of T cells, iNKT cells and iNKT subsets in tissue samples were compared between control subjects (32 samples used for total T-cell and iNKT-cell analysis, 23 samples used for iNKT subset analysis) and patients with CRSwNP (78 samples used for total T-cell and iNKT-cell analysis, 54 samples used for iNKT subset analysis). **(F)** The proportions of HLA-DR^+^PD-1^-^, HLA-DR^+^PD-1^+^, and HLA-DR^-^PD-1^-^ iNKT subsets in total iNKT cells from tissues of control (n = 23) and patient (n = 54) subjects. The Mann–Whitney test was used in comparison analysis in **(B, D)**; data presented in **(B, D)** as median with interquartile range; **p<0.01, ***p<0.001. ns, not significant.

We first analyzed the frequencies of iNKT cells in PBMCs obtained from patients with CRSwNP and healthy subjects. The percentages of iNKT cells among total T cells present in PBMCs were significantly increased in patients (ranging from 0.018% to 1.01%) than in healthy donors (ranging from 0.005% to 0.545%) (*p*<0.01) (**Figures 1A, B** **)**. iNKT cells can be subdivided into CD4^+^ and CD4^-^ subsets, in which CD4^-^ subsets usually suggest a Th1-biased cytokine activity compared to their CD4^+^ counterparts (28). CD4^+^ or CD4^-^ iNKT cells were comparable in PBMCs between patients and controls (**Figure 1B**). When evaluating the activation (HLA-DR) and exhaustion (PD-1) markers, the percentages of HLA-DR^+^ (3.71-fold) and PD-1^+^ (2.16-fold) iNKT cells were significantly higher in PBMCs from healthy donors than patients (**Figure 1B**); among these subsets, the double-positive (HLA-DR^+^PD-1^+^) iNKT cells were further increased by 6.04-fold (**Figure 1B**). HLA-DR^+^PD-1^+^ iNKT (39.04%) and HLA-DR^-^PD-1^+^ iNKT (28.91%) were the main subsets found among the total iNKT cells obtained from healthy subjects and patients, respectively (**Figure 1C**). However, no significant correlation was observed between the iNKT cell percentage and peripheral eosinophils, neutrophils, and monocytes found in CRSwNP patients (**Supplementary Figure 1**).

We then evaluated iNKT cells in tissues from patients with CRSwNP and control subjects. The patients exhibited a significant increase in the percentages of iNKT cells in polyp tissues (ranging from 0.019% to 2.2%) than in control tissues (0.04%–0.62%) (*p*<0.001) (**Figures 1D, E** **)**. Higher percentages of iNKT subsets, including CD4^+^ iNKT cells (1.95-fold), HLA-DR^+^ iNKT cells (1.48-fold), and PD-1^+^ iNKT cells (1.47-fold), were also observed in polyp tissues than in control mucosa (**Figure 1E**); moreover, HLA-DR^+^PD-1^+^ iNKT cells were also elevated (1.69-fold) in polyp samples (**Figure 1E**). Interestingly, the proportion of HLA-DR^+^PD-1^+^ iNKT cells appeared to be the main subset present in local tissues in both patients (51.51%) and controls (36.09%) (**Figure 1F**).

### Relationship Between iNKT Cells and Inflammatory Endotypes of CRSwNP

**Figure 2:**
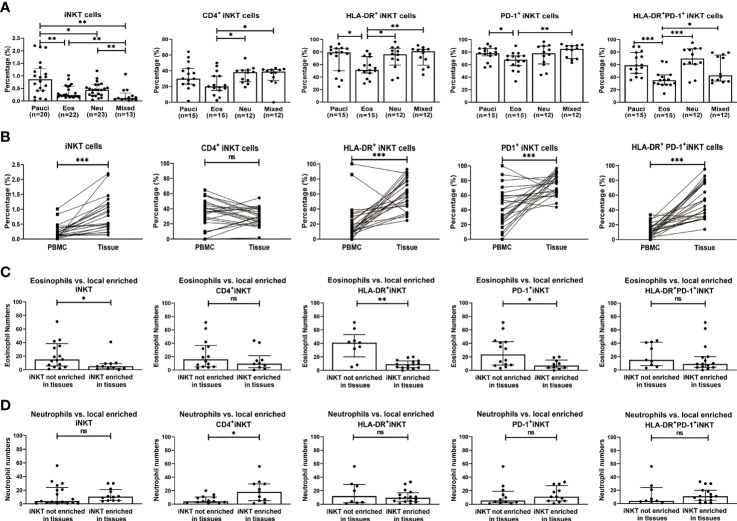
Relationship between percentages of iNKT cells and their subsets (CD4^+^, HLA-DR^+^, PD-1^+^, and HLA-DR^+^PD-1^+^ iNKT cells) and the infiltration patterns of inflammatory cells in CRSwNP. **(A)** Comparison of the percentages of iNKT cells and their subsets in paucigranulocytic, eosinophilic, neutrophilic, and mixed granulocytic polyps. **(B)** Comparisons of the percentages of iNKT cells and their subsets in tissues and matched PBMCs from patients with CRSwNP (n = 24). **(C)** Comparison of the tissue eosinophil counts in patients (n = 24) with high versus low local enrichment of total iNKT cells and their subsets. **(D)** Comparison of the tissue neutrophil counts in patients (n = 24) with high versus low local enrichment of total iNKT cells and their subsets. The Mann–Whitney test was used in comparison analysis in **(A, C, D)**; the Wilcoxon matched-pairs signed rank test was used in comparison analysis in **(B)**; data presented in **(A, C, D)** as median with interquartile range; *p<0.05, **p<0.01, ***p<0.001. ns, not significant.

The association of iNKT cells with eosinophilic/or neutrophilic polyps was investigated, and it was observed that the percentages of iNKT cells were negatively correlated with the numbers of eosinophils or neutrophils in polyp tissues (**Supplementary Figure 2**). Among the iNKT subsets, a positive correlation was found between CD4^+^ iNKT cells and neutrophils, while a negative correlation was identified between HLA-DR^+^ or HLA-DR^+^PD-1^+^ iNKT subsets and eosinophil infiltration (**Supplementary Figure 2**). The polyp tissues from patients with CRSwNP were further classified into four inflammatory phenotypes based on the local infiltration levels of eosinophils and neutrophils: paucigranulocytic (n=20), eosinophilic (n=22), neutrophilic (n=23), and mixed granulocytic (n=13). The results showed that percentages of iNKT cells were significantly lower in eosinophilic (0.23%) and mixed granulocytic (0.11%) polyps than in paucigranulocytic (0.86%) and neutrophilic polyps (0.49%) (*p*<0.01) (**Figure 2A**). Comparisons among the differential expressions of iNKT markers were further analyzed. CD4^+^ iNKT cells were increased in neutrophilic and mixed granulocytic polyps than in eosinophilic polyps, while polyps of the eosinophilic phenotype showed a significantly lower percentage of HLA-DR^+^ and PD-1^+^iNKT cells than those of paucigranulocytic or neutrophilic phenotypes (**Figure 2A**).

Finally, we evaluated the frequencies of iNKT cells in tissue samples and their matched PBMCs in patients with CRSwNP. The percentages of iNKT cells and subsets were significantly higher in tissues than in matched PBMCs (iNKT, 0.52% vs. 0.14%; HLA-DR^+^ iNKT, 61.35% vs. 13.35%; PD-1^+^ iNKT, 78.05% vs. 40%; HLA-DR^+^PD-1^+^ iNKT, 48.85% vs. 10.5%) (**Figure 2B**). We also compared cells obtained from patients that had locally enriched total iNKT cells or iNKT subsets (cell percentage in tissues divided by that in PBMCs, ratio ≥2) versus those from patients that did not show a local enrichment of such cells (ratio <2). Patients with a local enrichment of total iNKT cells and HLA-DR^+^ or PD-1^+^ iNKT subsets exhibited significantly lower eosinophil infiltration in polyps compared to those without local enrichment (**Figure 2C**). Approximately half of the patients showed an increase in the local enrichment of CD4^+^ iNKT cells. Patients with a higher local enrichment of the CD4^+^ iNKT subset (ratio ≥1) appeared to have an increase of the tissue neutrophil level than patients with a lower local enrichment of the CD4^+^ iNKT subset (ratio <1) (**Figure 2D**).

### Functional Subsets of iNKT Cells in Polyp Tissues and Their Association With Inflammatory Endotypes of CRSwNP

**Figure 3:**
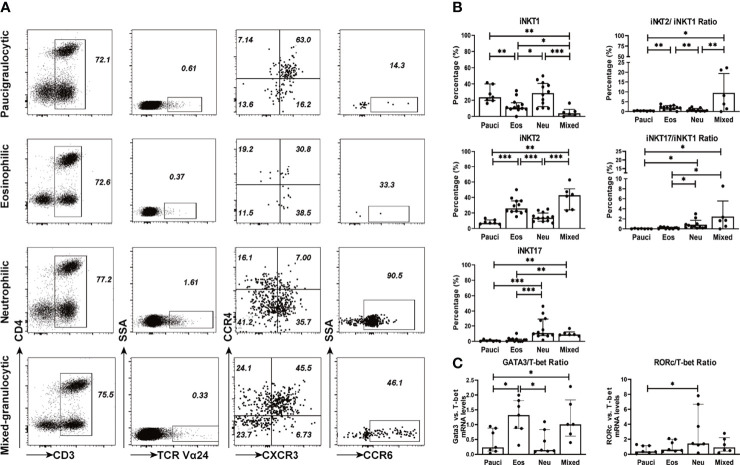
Functional subsets of iNKT cells in different inflammatory endotypes of CRSwNP. **(A)** Representative flow cytometric plots showed the frequencies of iNKT functional subsets based on characterization of surface markers, i.e., iNKT1 (CD3^+^ TCRVα24^+^ CXCR3^+^ CCR4^-^), iNKT2 (CD3^+^ TCRVα24^+^ CXCR3^-^ CCR4^+^), iNKT17 (CD3^+^ Vα24TCR^+^ CXCR3^-^ CCR4^-^ CCR6^+^). iNKT cells were identified through a subsequent gating strategy by lmyphocytes, CD3^+^ T cells, and TCR Va24 iNKT cells; the iNKT cells were further defined into iNKT subsets by using markers CXCR3, CCR4, and CCR6. Gates were set based on negative cell population, i.e., non-CD3^+^ T cells. **(B)** Comparisons of the percentages of iNKT1, iNKT2, and iNKT17 and the ratios of iNKT2/iNKT1 and iNKT17/iNKT1 in paucigranulocytic (n = 7), eosinophilic (n = 12), neutrophilic (n = 12), and mixed granulocytic (n = 6) polyp samples. **(C)** Comparisons of the ratios of GATA3/T-bet mRNA levels and RORc/T-bet mRNA levels in paucigranulocytic (n = 7), eosinophilic (n = 7), neutrophilic (n = 7), and mixed granulocytic (n = 6) polyp samples. The Mann–Whitney test was used in comparison analysis in **(B, C)**; data presented in **(B, C)** as median with interquartile range; *p<0.05, **p<0.01, ***p<0.001.

iNKT cells can be categorized into functionally distinct subsets (i.e., iNKT1, iNKT2, and iNKT17), which are distinguished by the expression of signature surface markers and transcription factors (8, 13, 29). Based on the characterization of surface markers, iNKT1 was defined as CD3^+^TCRVα24^+^CXCR3^+^CCR4^-^, iNKT2 was defined as CD3^+^TCRVα24^+^CXCR3^-^CCR4^+^, and iNKT17 was defined as CD3^+^TCRVα24^+^ CXCR3^-^CCR4^-^CCR6^+^. The percentages of iNKT2 cells were significantly higher in eosinophilic (25.95%) and mixed granulocytic polyps (42.80%) than in paucigranulocytic (7.14%) and neutrophilic (13.85%) phenotypes (*p*<0.001), while iNKT17 cells were increased in neutrophilic (10.76%) and mixed granulocytic (8.88%) polyps than in paucigranulocytic (0.73%) and eosinophilic groups (2.08%) (*p*<0.001). Furthermore, polyps with eosinophilic (11.10%) or mixed granulocytic (4.05%) phenotypes had lower frequencies of iNKT1 cells compared to paucigranulocytic (23.80%) or neutrophilic (28.70%) groups (*p*<0.01). Interestingly, mixed granulocytic polyps displayed the highest ratios of both iNKT2/iNKT1 cells and iNKT17/iNKT1 cells as compared to the other groups. (**Figures 3A, B** **)**. Correlation analysis also showed that the frequencies of iNKT2 and iNKT17 cells were positively associated with eosinophil and neutrophil infiltration, respectively, whereas the percentage of iNKT1 cells was negatively correlated with the eosinophil number (**Supplementary Figure 3**).

T-bet, GATA3, and RORc were determined in iNKT cells sorted from different inflammatory subtypes. The ratio of the GATA3/T-bet mRNA level was significantly increased in eosinophilic (5.58-fold) and mixed granulocytic (4.25-fold) polyps than in the paucigranulocytic group, while the ratio of the RORc/T-bet mRNA level was significantly upregulated in the neutrophilic polyps than in paucigranulocytic polyps (3.99-fold) (**Figure 3C**). The evaluation of both the surface markers and transcription factors demonstrated that iNKT2 and iNKT17 subsets were enriched in eosinophilic and neutrophilic mucosae, respectively.

### Comparisons Between iNKT Functional Subsets and Conventional Th Subsets

Conventional CD4^+^ Th subsets such as Th1 cells, CXCR3^+^CCR6^-^CCR4^-^; Th2 cells, CXCR3^-^CCR6^-^CCR4^+^; and Th17 cells, CXCR3^-^CCR4^-^CCR6^+^ were assessed using surface markers based on previous reports (30, 31). The proportion of iNKT and conventional CD4^+^ Th subsets among the total T cells was compared based on the surface marker expression (**Table 2** data presented as mean). The results showed the presence of higher levels of Th2 and Th17 subsets in eosinophilic and neutrophilic polyps, respectively, although the Th1 subset was the dominant population in each inflammatory endotype. Interestingly, unlike conventional CD4^+^ T cells, only the iNKT1 subset was predominantly found in paucigranulocytic polyps. The iNKT2 subset was dominant in eosinophilic or mixed granulocytic polyps, and a higher level of the iNKT17 subset was present in neutrophilic polyps than in other types of polyps. Furthermore, the ratios of iNKT2/iNKT1 and iNKT17/iNKT1 cells were higher than those of Th2/Th1 and Th17/Th1 cells, respectively. These results demonstrate that the functional skewing properties of iNKT subsets are more significant than those of conventional Th subsets.

**Table 2:** Comparisons between iNKT functional subsets and conventional Th subsets.

Cell types (% of total T cells)	Paucigranulocytic (n=7)	Eosinophilic (n=12)	Neutrophilic (n=12)	Mixed granulocytic (n=6)
iNKT1	0.34	0.07	0.24	0.05
iNKT2	0.10	0.16	0.10	0.21
iNKT17	0.01	0.02	0.14	0.07
iNKT2/iNKT1	0.34	1.96	0.82	9.47
iNKT17/iNKT1	0.04	0.17	0.82	2.46
Th1	16.10	8.43	14.24	10.88
Th2	2.60	12.99	6.37	11.90
Th17	0.11	0.27	0.88	1.19
Th2/Th1	0.24	2.61	0.69	1.80
Th17/Th1	0.01	0.05	0.09	0.19

a. iNKT and Th subsets are characterized by surface markers.

b. The percentages of iNKT and Th subsets are calculated by total T cells (CD3^+^).

c. The sample size is 37.

d. Data are presented as mean.

### Functionalities of iNKT Cells in Polyp Tissues

**Figure 4:**
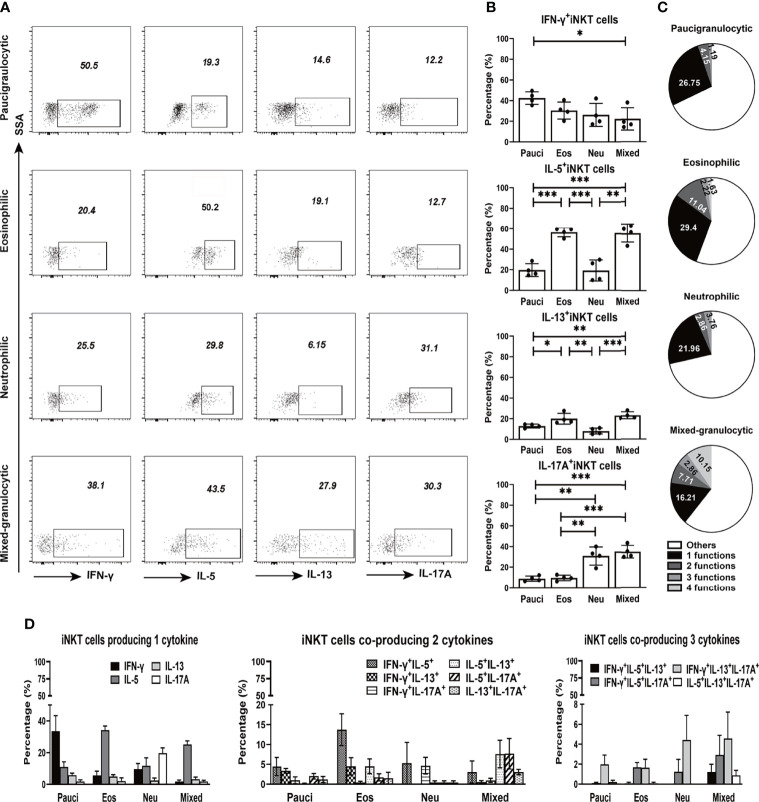
Cytokine production features of iNKT cells in different inflammatory endotypes of CRSwNP. **(A, B)** Representative flow cytometric pictures demonstrated the production of IFN-γ, IL-5, IL-13, and IL-17A in iNKT cells and their comparisons were analyzed in paucigranulocytic (n = 4), eosinophilic (n = 4), neutrophilic (n = 4), and mixed granulocytic (n = 4) polyp samples. **(C)** Boolean gating for cytokine analysis. Boolean gating was analysed in Flowjo through excluding and combine gating strategy. Pie charts showed the poly-functionality of iNKT cells in four different inflammatory subtypes of CRSwNP. **(D)** iNKT cells producing 1, 2, and 3 types of cytokines were compared in different inflammatory groups of CRSwNP. The unpaired t test was used in comparison analysis in **(B)**; data presented in **(B, D)** as mean with SD; *p<0.05, **p<0.01, ***p<0.001.

We further analyzed the ability of iNKT cells obtained from the polyps of different inflammatory endotypes to produce cytokines. iNKT cells derived from polyp tissues proliferated under the influence of IL-2 and αGalCer and were further assessed for intracellular cytokine levels (**Supplementary Figure 4**). Signature Th1 (IFN-γ), Th2 (IL-5 and IL-13), and Th17 (IL-17A) cytokines were measured in iNKT cells from four inflammatory subtypes of polyp tissues (**Figure 4A**). The percentages of type 2 iNKT cells were significantly increased in polyps with eosinophilic (IL-5^+^, 56.42%; IL-13^+^, 20.05%) or mixed granulocytic (IL-5^+^, 55.82%; IL-13^+^, 35.3%) patterns than in those with paucigranulocytic (IL-5^+^, 19.70%; IL-13+, 12.98%) (*p*<0.05) or neutrophilic (IL-5^+^, 19.30%; IL-13^+^, 8.11%) patterns (*p*<0.01). Moreover, the percentages of type 17 iNKT cells were significantly higher in neutrophilic (30.85%) or mixed granulocytic (35.30%) polyps than in those with paucigranulocytic (8.92%) or eosinophilic (9.63%) (*p*<0.01) ones (**Figure 4B**). These results confirm that iNKT cells from polyps with eosinophilia and neutrophilia are prone to produce high amounts of type 2 and type 17 cytokines, respectively.

Based on the ability of iNKT cells to produce IFN-γ, IL-5, IL-13, and IL-17A, the poly-functionality of local iNKT cells from different inflammatory endotypes of CRSwNP were analyzed. The results showed that a majority of iNKT cells expressed one type of cytokine, while iNKT cells in eosinophilic or mixed granulocytic polyps were more capable of producing two types of cytokines than those in paucigranulocytic or neutrophilic polyps; moreover, the mixed granulocytic phenotypic group was more competent in producing four cytokines than the other groups (**Figure 4C**). We further characterized the cytokine-producing profiles of iNKT cells of each inflammatory endotype. IFN-γ, IL-5, IL-17A, and IL-5 were the dominant cytokines produced by iNKT cells from paucigranulocytic, eosinophilic, neutrophilic, and mixed granulocytic polyps, respectively (**Figure 4D**). Regardless of the cosynthesis of two or three cytokines in iNKT cells, Th2 and Th17 cytokines represented the dominant types in eosinophilic and neutrophilic polyps, respectively (**Figure 4D**). Mixed granulocytic polyps appeared to show a high polyfunctional characteristic, while paucigranulocytic polyps were prone to have monofunctional properties related to cytokine production (**Figure 4D**).

### iNKT Cell Functional Skewing Regulated by Polyp Tissue Microenvironment

**Figure 5:**
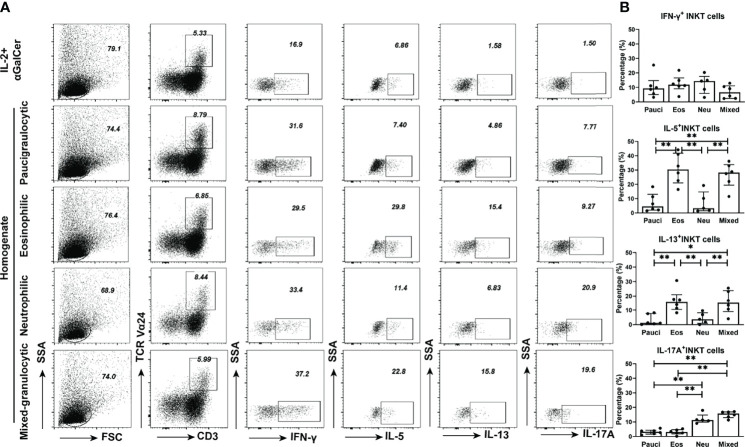
Functional properties of iNKT cells in PBMCs stimulated by different inflammatory endotypes of CRSwNP. **(A)** Representative flow cytometric pictures showed the production of IFN-γ, IL-5, IL-13, and IL-17A in iNKT cells from PBMCs which were stimulated by different inflammatory endotypes of polyp tissues. **(B)** Comparisons of IFN-γ^+^, IL-5^+^, IL-13^+^, and IL-17A^+^ iNKT cells from PBMCs stimulated by tissue homogenates from paucigranulocytic (n = 6), eosinophilic (n = 6), neutrophilic (n = 5) and mixed granulocytic (n = 6) polyps. The values in **(B)** were subtracted by the cytokine level measured in the cells incubated with IL-2 plus αGalCer. The Mann–Whitney test was used to analyze the statistical difference in **(B)**; data presented in **(B)** as median with interquartile range; *p<0.05, **p<0.01.

The functional characteristics of iNKT cells can be modified by the local microenvironment (particularly *via* certain cytokine factors) (14, 18). Homogenates from different inflammatory endotype polyps were used to stimulate the PBMCs from healthy donors, and the cytokine production patterns of iNKT cells were evaluated. Tissue homogenates (at concentrations of 500 or 2,500 ng/ml) from all inflammatory groups of polyps could significantly promote the proliferation of iNKT cells (**Supplementary Figure 5**). Polyp homogenates from eosinophilic or mixed granulocytic mucosae significantly enhanced the proportions of IL-5^+^ iNKT cells and IL-13^+^ iNKT cells in PBMCs, and homogenates from neutrophilic or mixed granulocytic polyps had significant effects on the ability of IL-17A^+^ iNKT cells to cause the skewing of PBMCs. Furthermore, the homogenates from different inflammatory types of polyps exhibited similar effects on IFN-γ production in iNKT cells (**Figures 5A, B**).

We then investigated which cytokine factors contribute to the functional skewing of iNKT cells in polyp homogenates. Three cytokines (IL-15, IL-7, and IL-23) critical for iNKT functional development were selected for the measurement of different inflammatory endotypes of tissue homogenates (32–34). The results showed that the levels of IL-7 and IL-23 were significantly upregulated in eosinophilic and neutrophilic polyps, respectively, than in the other subtypes of polyps, while IL-15 concentrations were comparable in all inflammatory groups (**Supplementary Figure 6**). Hence, we further analyzed whether Th2- or Th17-skewing by iNKT cells in eosinophilic and neutrophilic tissue microenvironments could be regulated by IL-7 and IL-23. PBMCs were first stimulated with eosinophilic or neutrophilic tissue homogenates, and the downregulation of GATA3 and RORc mRNA levels was found in PBMCs treated with antibodies to IL-7 and anti-IL-23, respectively (**Supplementary Figure 7**). These results confirm that antibodies to IL-7 and anti-IL-23 can reduce the polyp homogenate induced-Th2 and -Th17 responses in PBMCs.

**Figure 6:**
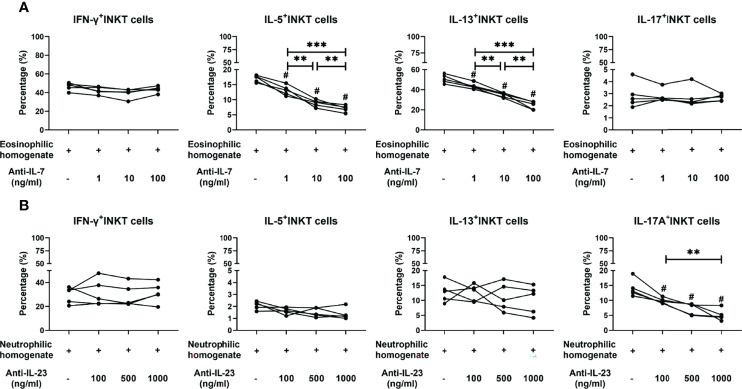
Effects of neutralizing antibodies on cytokine production features of iNKT cells stimulated by tissue homogenates. **(A)** Percentages of IFN-γ^+^, IL-5^+^, IL-13^+^, and IL-17A^+^ iNKT cells were compared in iNKT cells treated with homogenates from eosinophilic polyps (n=5) and anti-IL-7 antibody at concentrations of 1, 10, and 100 ng/ml. **(B)** Percentages of IFN-γ^+^, IL-5^+^, IL-13^+^, and IL-17A^+^ iNKT cells were compared in iNKT cells treated with homogenates from neutrophilic polyps (n = 5) and anti-IL-23 antibody at concentrations of 1, 10, and 100 ng/ml. Paired t test was used in the above comparison analysis; data presented in **(A, B)** as mean; ^#^p<0.05 versus homogenate control; **p<0.01, ***p<0.001.

Next, the cytokine-producing properties of iNKT cells in PBMCs upon treatment with homogenates and antibodies were analyzed. When using an IL-7 antibody, iNKT cells treated with eosinophilic polyp homogenates showed a decreased capacity to produce IL-5 and IL-13 in a dose-dependent manner, whereas treatment with IL-7 antibody did not affect IFN-γ and IL-17A production (**Figure 6A** and **Supplementary Figure 8**). When an antibody to IL-23 was used, iNKT cells stimulated with neutrophilic polyp homogenates presented a reduced production of IL-17A in a dose-dependent manner; however, blocking the effect of IL-23 appeared to have no impact on the production of IFN-γ, IL-5, and IL-13 in iNKT cells (**Figure 6B** and **Supplementary Figure 9**). These data suggest that the eosinophilic and neutrophilic microenvironments of polyp mucosa can favor iNKT cells to develop type 2- and type 17-bias functional subsets, and that these effects are partially dependent on IL-7 and IL-23 in local tissues, respectively.

## Discussion

Due to an incomplete understanding of human iNKT cell subsets, only the presence of total iNKT cells in various tissues (including airway mucosa) has been described (22, 24). To the best of our knowledge, this study is the first to demonstrate the frequencies of iNKT cells and their functional subsets in both the tissues and PBMCs obtained from patients with chronic airway inflammation (like CRSwNP). Moreover, it reveals the relationship between iNKT cells and inflammatory endotypes of CRSwNP and elucidates the effects of different inflammatory microenvironments on the functional development of iNKT cells.

Our results showed that the range of iNKT cells in polyp tissues is from 0.019% to 2.2%, which is similar to the previous report showing a range of 0%–2.38% of iNKT cells in polyp samples from Caucasian patients (24). We noticed that the local iNKT cells display higher proportions of HLA-DR^+^ and PD-1^+^, with the coexpression of the HLA-DR^+^ PD-1^+^ phenotype in the chronic inflammatory airway mucosa. Such phenomenon suggests that an activation status (HLA-DR^+^) of iNKT cells, which may lead to an increased exhaustion of (PD-1^+^) iNKT cells during chronic inflammation. Similar findings were reported in some tumor studies, which also showed highly activated (HLA-DR^+^ CD38^+^ PD-1^+^) phenotypes (35). Furthermore, the local enrichment of iNKT cells is negatively related to the number of eosinophils, which further suggest that the eosinophilic microenvironment may impact the chemo-attractants of iNKT cells to the local mucosa.

A unique ability of iNKT cells is to promptly produce multiple effector cytokines upon stimulation by various pathogens or inflammatory mediators in local tissues. It has been recognized that iNKT cells represent a heterogeneous population with several functional subsets. Our results showed that the proportion of type 1 iNKT cells was high in paucigranulocytic polyps. Several reports have indicated that a higher eosinophil infiltration in polyps is correlated with polyp recurrence (36), while neutrophilic polyps are related to steroid resistance in Asian patients (37). iNKT1 is the primary producer of IFN-γ, which can balance the effects of type 2 iNKT cells in nasal mucosal inflammation. Thus, we speculate that the increasing iNKT1 proportion might be related to a less severe type of polyps, indicating the potential protective role of this functional subset in the nasal mucosa. In contrast, the enrichment of type 2 and type 17 iNKT subsets in eosinophilic and neutrophilic polyps suggests that the pathogenic functions of these two iNKT subsets contribute to the inflammation of the polyp mucosa. It is well known that iNKT cells develop in the thymus, and our results demonstrated that a particular microenvironment may further shape the cytokine production capability of iNKT cells. In our study, eosinophilic polyps with high IL-7 levels and neutrophilic polyps with high IL-23 levels could influence iNKT cells to produce IL-5 and IL-17, respectively, most likely *via* the activation of IL-7Rα and IL-23R receptors present on iNKT cells. Other than cytokine factors, iNKT cells can recognize lipid antigens that could be derived from foreign pathogens. Since the nasal mucosa is frequently contacting environmental pathogens, certain bacteria-derived lipid antigens might activate iNKT subsets and potentially functionally skew these cells.

Mouse models are widely used to study the roles of iNKT cells in airway inflammation, such as asthma. Mice deficient in CD1d^-/-^ and Jα18^-/-^ iNKT cells showed a decrease in airway hyperresponsiveness and eosinophilia, while the adoptive transfer of IL-4- and IL-13-producing iNKT cells restored the airway inflammation (38, 39), suggesting that type 2 iNKT cells are responsible for asthmatic inflammation (20, 40). In addition, IL-17-producing iNKT cells can recruit neutrophils into the lungs (21), and can also cause disease severity in asthmatic mice induced by air pollutants (e.g., ozone) (41). Taking into account the studies performed on the mechanism of action of iNKT cells in asthma models and the rapid immune response of these cells during the initial stage of inflammation, we speculate that enrichment of type 2 and type 17 iNKT cells in the nasal mucosa may be responsible for priming the immune system to Th2-biased and Th17-biased phenotypes in CRSwNP inflammation. Nevertheless, it is worthwhile to further investigate whether iNKT cells have direct or indirect effects on the activation of Th2 cells, eosinophils, and neutrophils in chronic inflammatory nasal mucosa.

In the current study, we compared the relative frequencies of iNKT functional subsets and conventional CD4^+^ Th subsets. The absolute proportions of Th1, Th2, and Th17 cells among total T cells were observed to be higher than those of iNKT1, iNKT2, and iNKT17 cells; moreover, the functional skewing properties were also found to be higher. iNKT subsets have more “unique” functions than conventional Th subsets in distinct inflammatory endotypes of polyps. Future studies to directly compare the contribution of iNKT subsets and conventional Th subsets to different inflammatory patterns of chronic nasal inflammation are worth performing.

Since there are limited numbers of iNKT cells present in tissues and nasal/bronchial secretions obtained from patients (22), it is difficult to characterize the functional properties by directly sorting specific iNKT subsets (iNKT1, iNKT2, and iNKT17) from mucosal tissues. Hence, using the state-of-art techniques such as single-cell RNAseq would be a feasible approach to reveal more detailed information on iNKT functional subsets in the future.

Our study demonstrates a functional relationship between local iNKT cell subsets and distinct inflammatory endotypes of CRSwNP and suggests that different types of inflammatory microenvironments in polyps could regulate the functional skewing of iNKT cells. Clarifying the functional diversity within iNKT cells in chronic airway inflammation (such as CRSwNP) is fundamental for developing novel therapeutic strategies to target specific subsets and manipulate immune responses in inflammatory nasal mucosa.

## Data Availability Statement

The original contributions presented in the study are included in the article/**Supplementary Material**. Further inquiries can be directed to the corresponding authors.

## Ethics Statement

The studies involving human participants were reviewed and approved by the First Affiliated Hospital, Sun Yat-sen University. The patients/participants provided their written informed consent to participate in this study.

## Author Contributions

CL and YG conceived the project, designed research studies, reviewed the data, and wrote the manuscript. XY and QB conducted the experiment, acquired data, analyzed results, and contributed to writing the manuscript. HC, QM, and QL recruited patients and collected clinical samples. LS, WH, LJ, and BF performed the experiment. JL, WL, and WW commented on the manuscript.

## Funding

This study was supported by grants from the National Natural Science Foundation of China (81974139, 81974141, 81770983, 31800758, 32070882) and Guangzhou Science and Technology Programme (201907010038).

## Conflict of Interest

The authors declare that the research was conducted in the absence of any commercial or financial relationships that could be construed as a potential conflict of interest.

## Publisher’s Note

All claims expressed in this article are solely those of the authors and do not necessarily represent those of their affiliated organizations, or those of the publisher, the editors and the reviewers. Any product that may be evaluated in this article, or claim that may be made by its manufacturer, is not guaranteed or endorsed by the publisher.
