# Supplementary material for: Type 2 and Type 17 Invariant Natural Killer T Cells Contribute to Local Eosinophilic and Neutrophilic Inflammation and Their Function Is Regulated by Mucosal Microenvironment in Nasal Polyps
Source: Front Immunol. 2022 Jun 3;13:803097. doi: 10.3389/fimmu.2022.803097 (PMC9204195; doi:10.3389/fimmu.2022.803097)
Supplement: Supplementary file 1 [file DataSheet_1.docx]

**Supplementary materials**

**Table 1** Summary of the number of sample materials used in experimental assays

| **Materials and experiments** | **CRSwNP patients** |
| --- | --- |
| Histological evaluations in FFPE samples | 80 |
| Flow cytometry analysis in PBMCs | 33 |
| - Total iNKT cells | 33 |
| - iNKT subsets (by CD4, HLA-DR, PD-1) | 28 |
| Flow cytometry analysis in tissues | 78 |
| - Total iNKT cells | 78 |
| - iNKT subsets (by CD4, HLA-DR, PD-1) | 54 |
| - iNKT functional subsets (iNKT1, iNKT2, iNKT17) | 37 |
| - Cytokine production in iNKT cells | 16 |
| Quantitative RT-PCR in tissue-isolated iNKT cells | 27 |
| Homogenates used for *in vitro* PBMC stimulation | 23 |
| ELISA assay in tissue homogenates | 16 |
| Homogenates used for *in vitro* PBMC stimulation with neutralizing antibodies | 10 |

**Table 2** Antibody information

| **Target antigen** | **Species** | **Clone** | **Conjugate** | **Brand** |
| --- | --- | --- | --- | --- |
| CD3 | Mouse | UCHT1 | Percp cy5.5 | Biolegend |
| CD3 | Mouse | BW264/56 | Percp cy5.5 | Milteny Biotec |
| CD4 | Mouse | SK3 | BV510 | BD |
| TCR Vα24 | Mouse | 6B11 | PE | Milteny Biotec |
| HLA-DR | Mouse | L243 | APC CY7 | Biolegend |
| PD-1 | Mouse | EH12.2H7 | BV421 | Biolegend |
| CCR4 | Mouse | L291H4 | BV421 | Biolegend |
| CXCR3 | Mouse | G025H7 | APC CY7 | Biolegend |
| CCR6 | Mouse | G034E3 | PE CY7 | Biolegend |
| IFN-γ | Mouse | B27 | APC CY7 | Biolegend |
| IL-5 | Rat | JES1-39D10 | FITC | Milteny Biotec |
| IL-13 | Rat | JES10-5A2 | PE CY7 | Biolegend |
| IL-13 | Mouse | eBio13A | PE CY7 | Invitrogen |
| IL-17A | Mouse | N49-653 | AF700 | BD |

**Table 3** Primers used for quantitative RT-PCR

| **Gene** | **Application** | **Primer sequences** |
| --- | --- | --- |
| GAPDH | qPCR | F: 5’-GGGAAGCTTGTCATCAATGGAA-3’ |
|  |  | R: 5′‐AGAGATGATGACCCTTTTGGCTC‐3′ |
| T-bet | qPCR | F: 5′‐CAACGCTTCCAACACGCAT‐3′ |
|  |  | R: 5′‐GACTCAAAGTTCTCCCGGAA‐3′ |
| GATA3 | qPCR | F: 5′‐TCATTAAGCCCAAGCGAAGG‐3′ |
|  |  | R: 5′‐GTCCCCATTGGCATTCCTC‐3′ |
| RORc | qPCR | F: 5′‐GCAGCGCTCCAACATCTTCT‐3′ |
|  |  | R: 5′‐ACGTACTGAATGGCCTCGGT‐3′ |
| GAPDH | pre-amplification | F: 5′‐ATGGCAAATTCCATGGCACCGTC‐3′ |
|  |  | R: 5′‐CGGCCATCACGCCACAGTT‐3′ |
| T-bet | pre-amplification | F: 5′‐CACTGGATGCGCCAGGAAGT‐3′ |
|  |  | R: 5′‐ATCCTTCGCCTGGCCAGGA‐3′ |
| GATA3 | pre-amplification | F: 5′‐GGCTTCGGATGCAAGTCCA‐3′ |
|  |  | R: 5′‐GTTTCTGGTCTGGATGCCTTCC‐3′ |
| RORc | pre-amplification | F: 5′‐CCCAGTTTCCGCAGCACAC‐3′ |
|  |  | R: 5′‐CCGTGCGGTTGTCAGCATT‐3′ |

**Supplementary figure legends**

**Figure 1** Correlation analysis of peripheral iNKT cells with levels of peripheral eosinophils, neutrophils, and monocytes in CRSwNP patients (n=33). Spearman correlation was used to analyze the correlation coefficient value (r value) and the statistical significance level. The scales of X and Y axis was transformed to logarithms.

**Figure 2** Correlation analyses of iNKT cells (n=78) and their subsets (CD4+, HLA-DR+, PD-1+, and HLA-DR+PD-1+ iNKT cells) (n=54) with levels of eosinophils and neutrophils in tissues of CRSwNP patients. Spearman correlation was used to analyze the correlation coefficient value (r value) and the statistical significance level. The scales of X and Y axis was transformed to logarithms.

**Figure 3** Correlation analyses of percentages of iNKT functional subsets (iNKT1, iNKT2, iNKT17, ratio of iNKT2/iNKT1, ratio of iNKT17/iNKT1) with levels of eosinophils and neutrophils in tissues of CRSwNP patients (n=37). Spearman correlation was used to analyze the correlations coefficient value (r value) and the statistical significance level. The scales of X and Y axis was transformed to logarithms.

**Figure 4** Flow cytometric pictures showed proliferation status of iNKT cells with no stimulation and incubation of IL-2 or IL-2 plus αGalCer.

**Figure 5** Proliferation of iNKT cells in PBMCs by stimulation of tissue homogenates. PBMCs from healthy donors were stimulated by IL-2 only, IL-2 plus αGalCer, and IL-2 plus αGalCer and homogenates, respectively. (A) Percentages of iNKT cells were analyzed by flow cytometry. (B) iNKT cells were compared in IL-2 plus αGalCer treated cells with and without stimulation of homogenates. Paired t test was used in the comparison analysis. # p<0.05 versus IL-2 plus αGalCer group; *p<0.05, **p<0.01.

**Figure 6** Concentrations of IL-15, IL-7, and IL-23 in polyp homogenates. The levels of IL-15, IL-7, and IL-23 in tissue homogenates from paucigranulocytic (n=4), eosinophilic (n=4), neutrophilic (n=4) and mixed granulocytic (n=4) polyps were detected by ELISA. Paired t test was used to analyze the statistical difference; data presented as mean with SD; *p<0.05, **p<0.01.

**Figure 7** Effects of neutralizing antibodies on expression of transcription factors in PBMCs stimulated by tissue homogenates. (A) PBMC samples were treated with eosinophilic homogenates with and without anti-IL-7 antibody at concentrations of 1, 10, and 100 ng/ml; and mRNA levels of T-bet, GATA3, and RORc were determined by quantitative RT-PCR. (B) PBMC samples were treated with neutrophilic homogenates with and without anti-IL-23 antibody at concentrations of 100, 500, and 1000 ng/ml; and mRNA levels of T-bet, GATA3, and RORc were determined by quantitative RT-PCR. Paired t test was used in the comparison analysis; # p<0.05 versus homogenate control; *p<0.05.

**Figure 8** Representative flow cytometric plots of IFN-γ, IL-5, IL-13, and IL-17A expression in iNKT cells treated with IL-2 plus αGalCer, IL-2 plus αGalCer and eosinophilic polyp homogenates with or without anti-IL-7 antibody (at concentrations of 1, 10, and 100 ng/ml).

**Figure 9** Representative flow cytometric plots of IFN-γ, IL-5, IL-13, and IL-17A expression in iNKT cells treated with IL-2 plus αGalCer, IL-2 plus αGalCer and neutrophilic polyp homogenates with or without anti-IL-23 antibody (at concentrations of 100, 500, and 1000 ng/ml).
