# Supplementary figures and images for: Type 2 and Type 17 Invariant Natural Killer T Cells Contribute to Local Eosinophilic and Neutrophilic Inflammation and Their Function Is Regulated by Mucosal Microenvironment in Nasal Polyps
Source: Front Immunol. 2022 Jun 3;13:803097. doi: 10.3389/fimmu.2022.803097 (PMC9204195; doi:10.3389/fimmu.2022.803097)

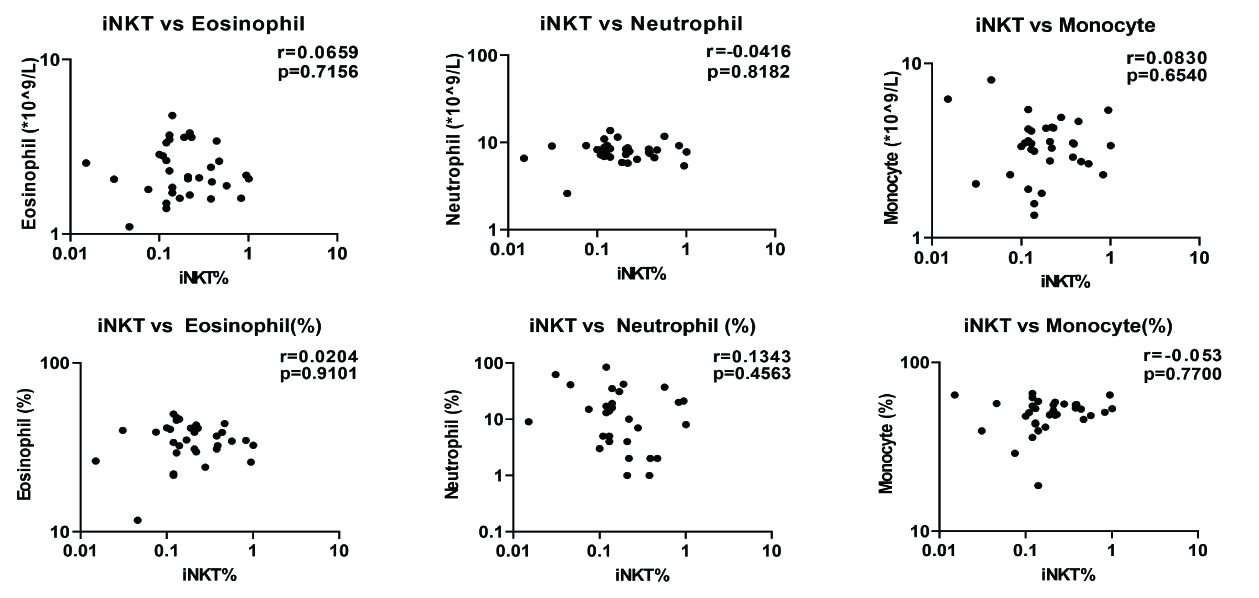

Supplement: Supplementary file 2 [file Image_1.tif]

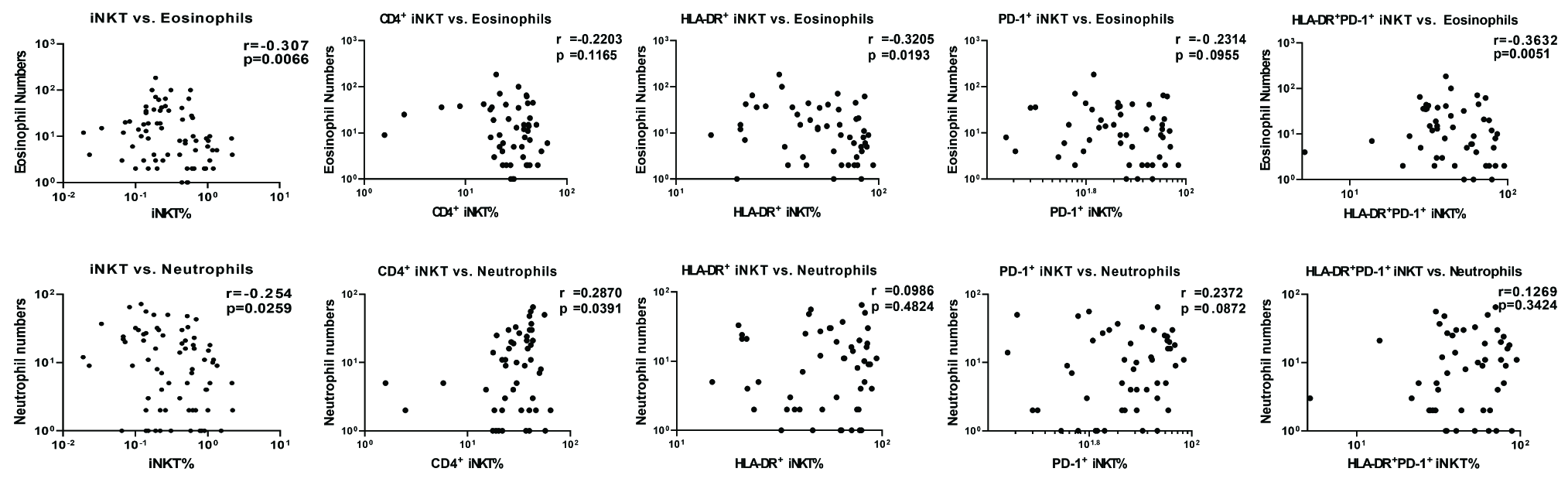

Supplement: Supplementary file 3 [file Image_2.tif]

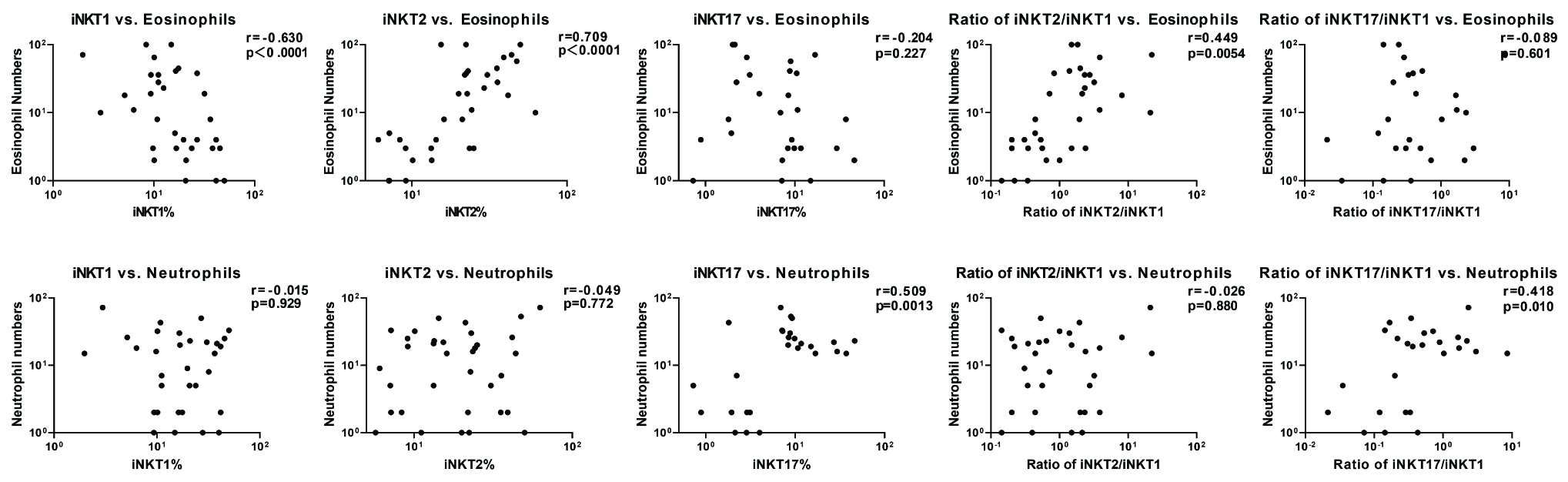

Supplement: Supplementary file 4 [file Image_3.tif]

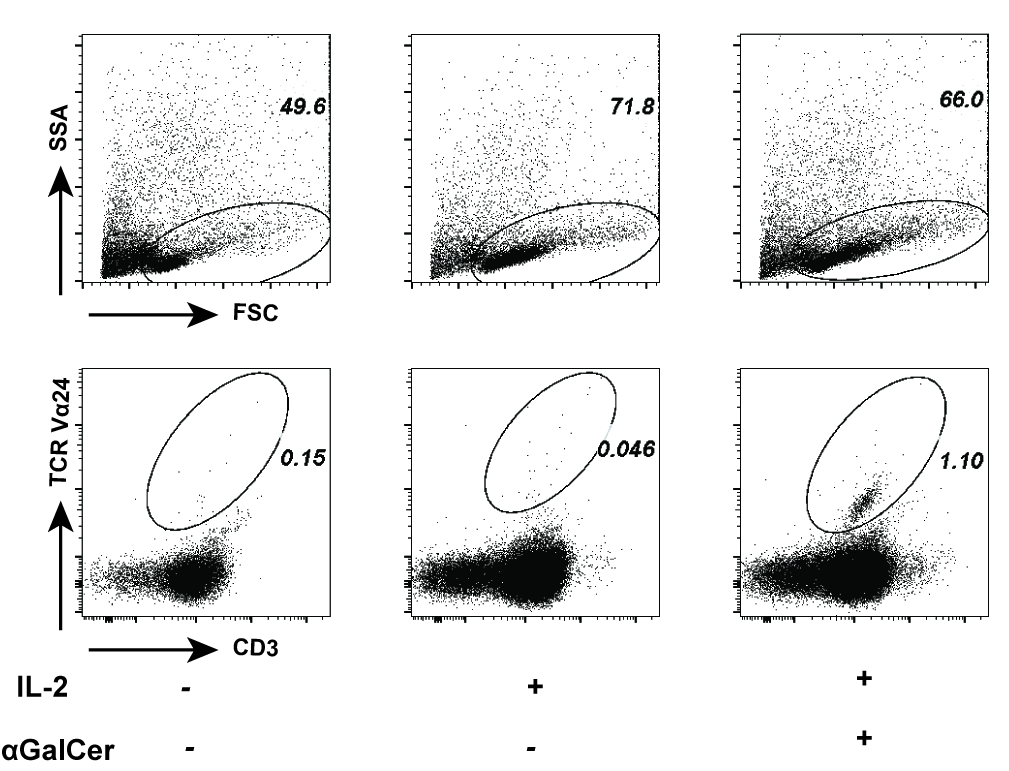

Supplement: Supplementary file 5 [file Image_4.tif]

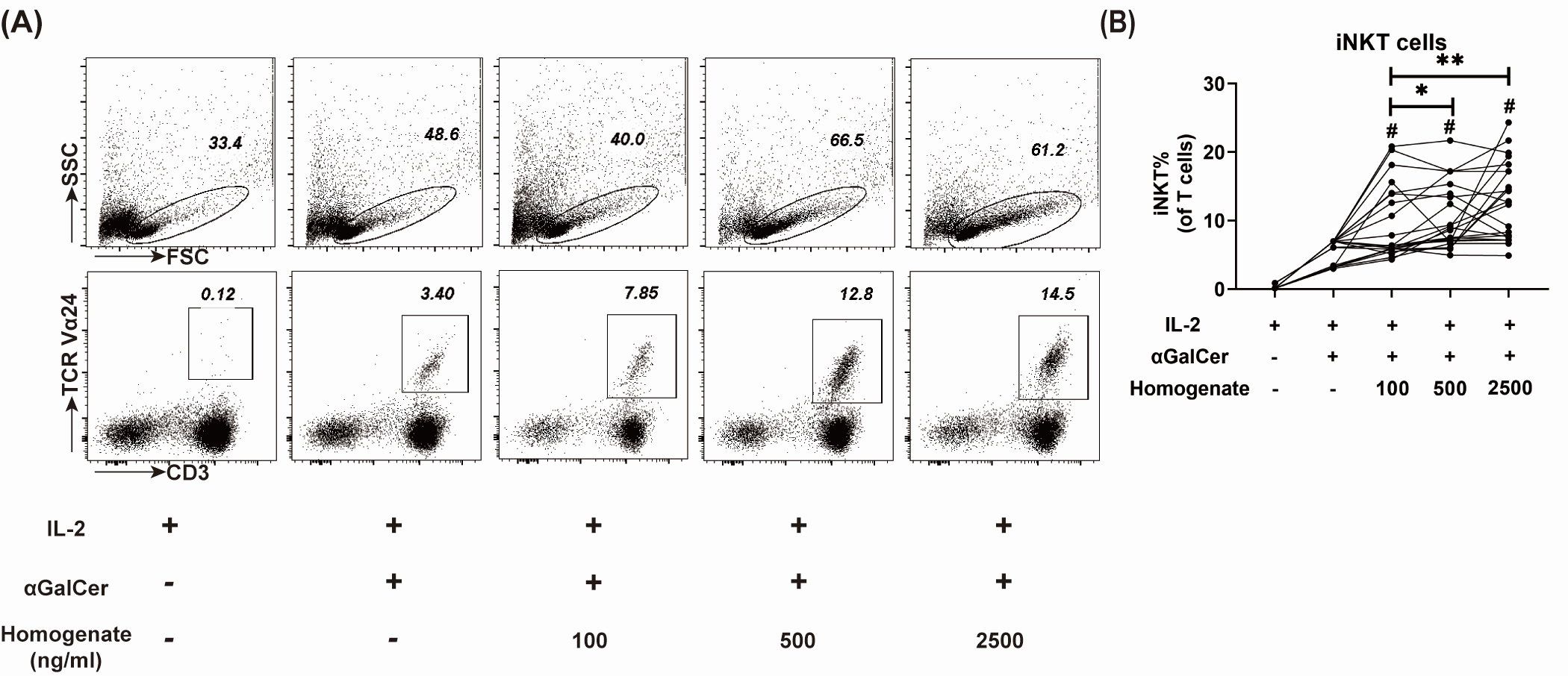

Supplement: Supplementary file 6 [file Image_5.jpeg]

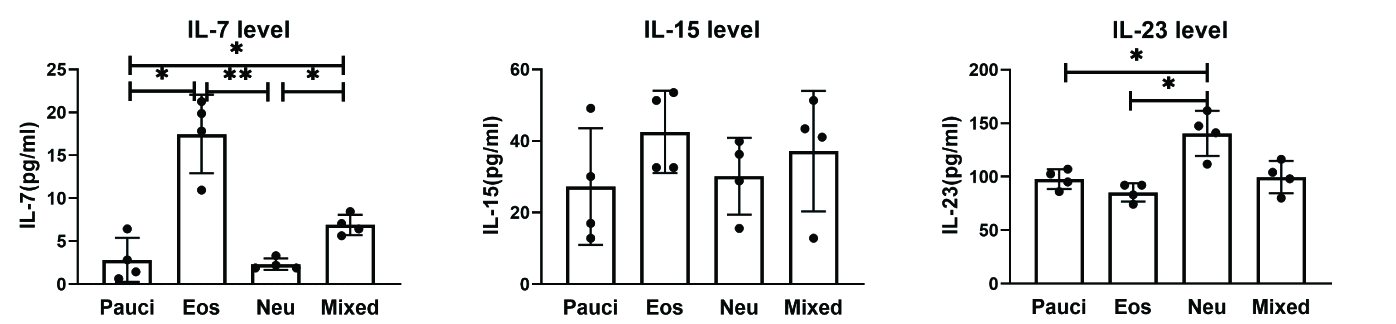

Supplement: Supplementary file 7 [file Image_6.tif]

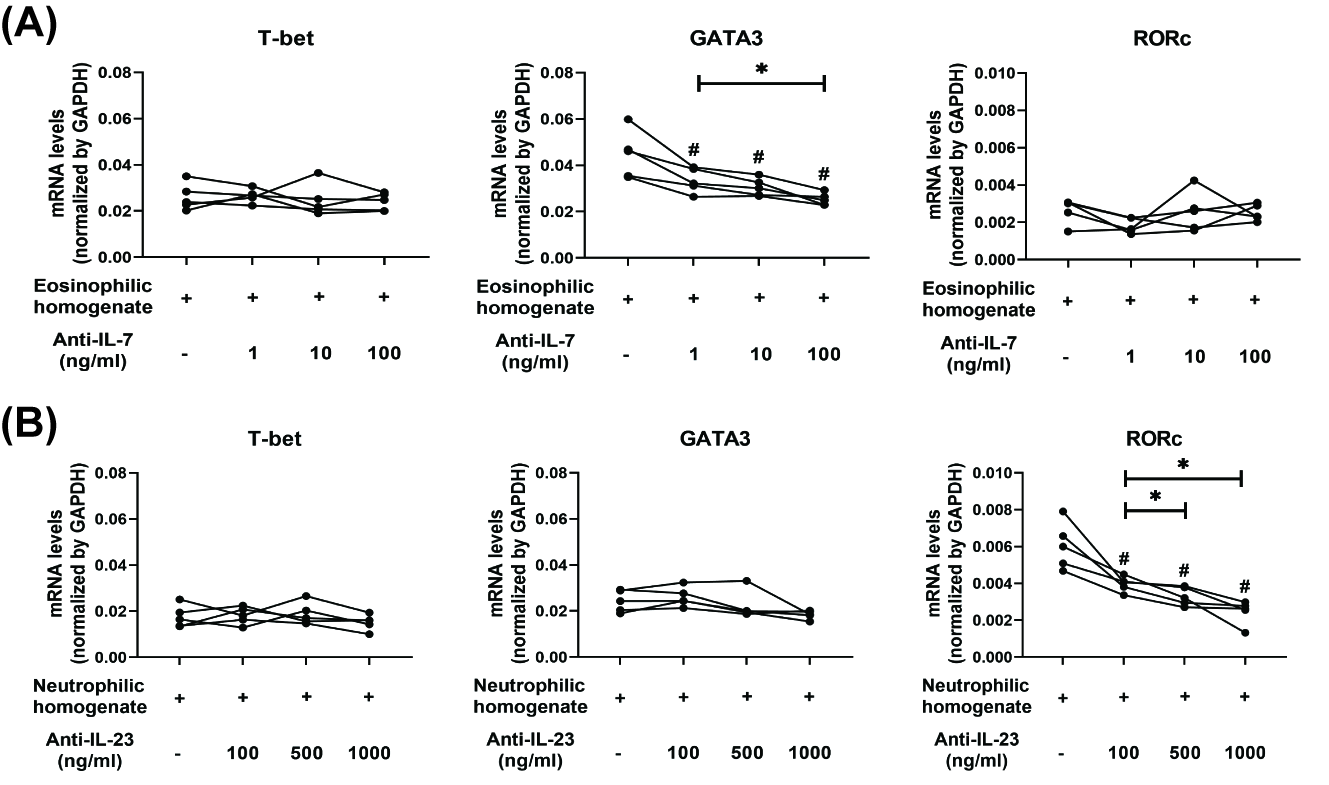

Supplement: Supplementary file 8 [file Image_7.tif]

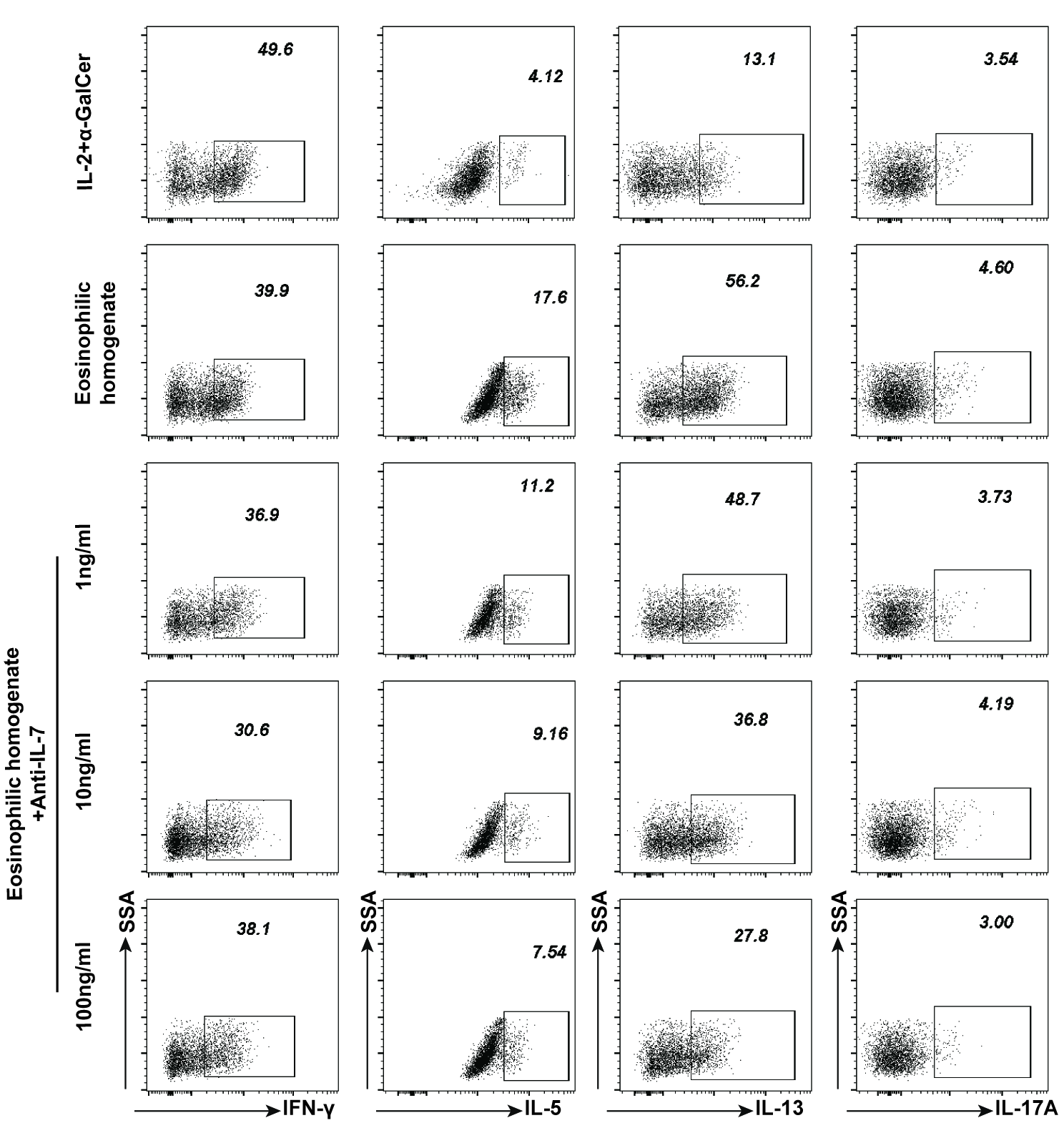

Supplement: Supplementary file 9 [file Image_8.tif]

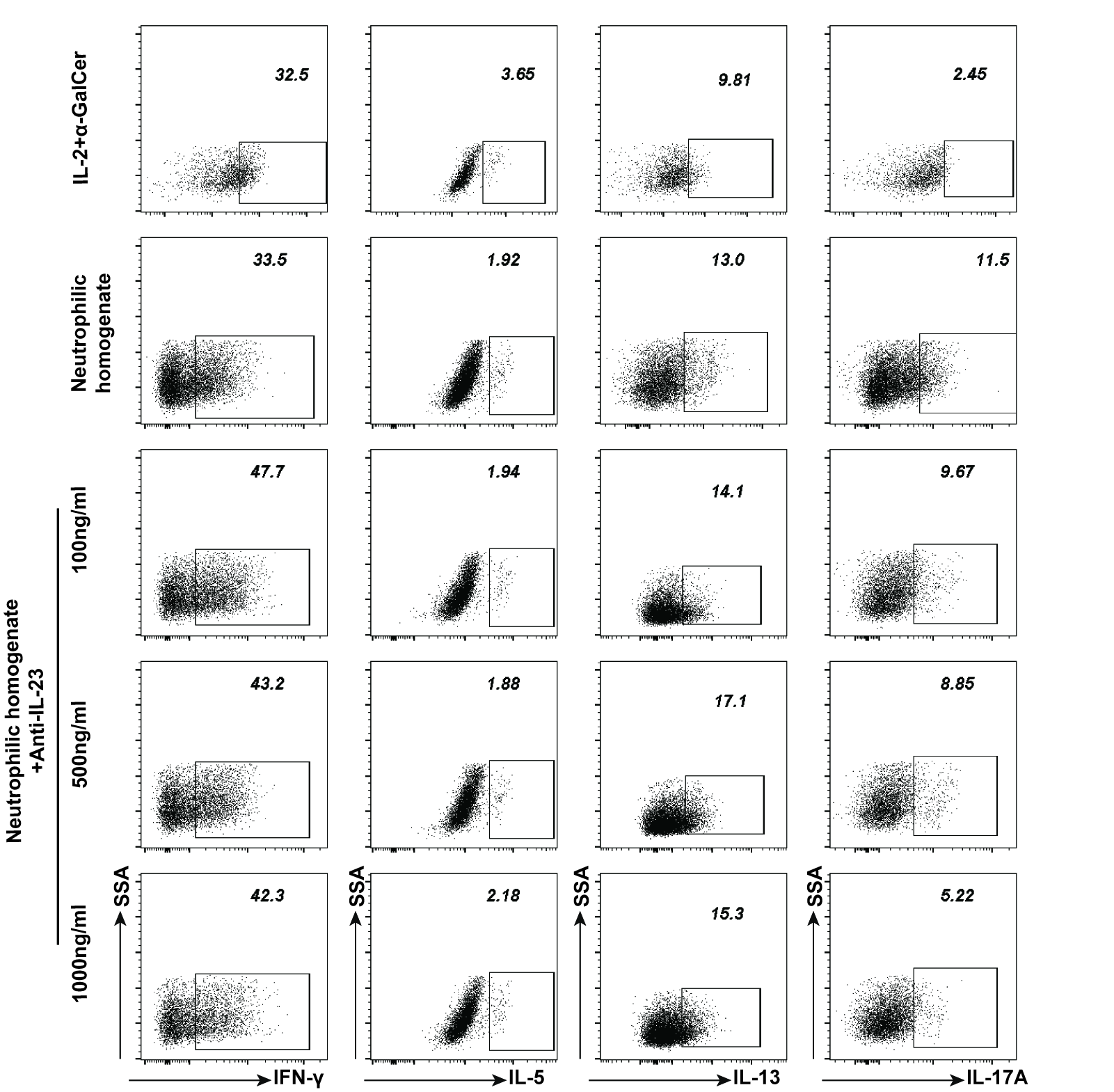

Supplement: Supplementary file 10 [file Image_9.tif]
